# Effectiveness of the Responding to Experienced and Anticipated Discrimination (READ) training on reducing stigma for medical students in Tunisia

**DOI:** 10.1371/journal.pone.0352158

**Published:** 2026-06-29

**Authors:** Lamia Jouini, Uta Ouali, Yosra Zgueb, Emna Bouguira, Ioannis Bakolis, Fethi Nacef, Claire Henderson

**Affiliations:** 1 Department of Psychiatry A of Razi Hospital, Faculty of Medicine of Tunis, University Tunis El Manar, Tunis, Tunisia; 2 Department of Biostatistics and Health Informatics, King’s College London Institute of Psychiatry, Psychology & Neuroscience, London, United Kingdom; 3 Health Service and Population Research Department, King’s College London Institute of Psychiatry, Psychology & Neuroscience, London, United Kingdom; University of Pisa, ITALY

## Abstract

Doctors have been identified as having a crucial role in responding to anticipated and experienced stigma of People with Mental Illness (PWMI). This paper aims to evaluate the effectiveness of the READ (Responding to Experienced and Anticipated Discrimination), an anti-stigma training for medical students, by measuring changes in their knowledge, attitudes, and skills, in responding to patients anticipated and experienced discrimination. The Mental Health Knowledge Schedule (MAKS), the Mental Illness Clinicians’ Attitudes version 2 (MICA2), and an OSCE (Observed Structured Clinical Examination) were used to determine participants’ knowledge, attitudes, and behaviours towards PWMI before and immediately after the training. There was evidence of difference in MICA2 mean total scores in the intervention group were compared to the control group after adjusting for age, gender and MICA baseline mean total scores (MD: −7.88; p < 0.001; 95% CI: −10.23 to −3.96). Moreover, the intervention group was 4.45 times more likely to be scored “pass” in the OSCE compared to the control group (p = 0.046, 95% CI: 1.03 to 19.26) after adjusting for age, gender and OSCE baseline scores. The positive changes in students’ attitudes and skills after the READ training should encourage further research on the causal pathways of this positive relationship.

## Introduction

People With Mental Illness (PWMI) can be subjected to stigma and discrimination from health professionals working in different fields [1–3]. Experiences of discrimination have been reported by 31% of service users in physical-health care settings and 44% of service users in mental-health care settings [4]. A review by Henderson et al conducted in 2014 [1] found that professionals suffering from burnout, professionals working in mental health and early career professionals are the ones most in need of interventions to decrease their stigmatization of patients.

A plethora of research investigated the effect of different approaches to reduce the stigma of mental illness. Interventions were mainly divided into three categories: education, social contact and social activism [5]. In a scoping review on interventions aiming to decrease health students ‘stigma towards patients with schizophrenia by Chen & all conducted in 2024 [6], interventions ranged from theoretical lessons, movies, role-playing, reading and discussion to drawing, focus group discussions, contact with PWMI through internships and augmented reality. Duration of interventions varied from a 45-minute voice simulation experience to a 13-week program. Overall, diverse interventions have shown potential to shift attitudes toward schizophrenia, though their effectiveness varies depending on the approach and context. Among all types of interventions, a combination of education and social contact, was shown to be the most effective approaches for specific target groups such as medical students [6, 7]. However, these studies were subject to many methodological limitations, mainly the lack of assessment of their sustainability on the long-term, the focus on High-Income Countries (HIC) [8], and the considerable heterogeneity in interventions, controls and outcome measurements across studies [9–12]. Furthermore, few studies have specifically evaluated stigma-reduction interventions among medical students using measures adapted to this population and the majority of studies focused on assessing knowledge and attitudes while neglecting behavioural outcomes [7, 9, 13].A recent review of the literature by Heim et al in 2019 [9], focusing on mental-health related stigma reduction interventions among medical and nursing students in LMIC, reported significant improvements in outcome measures, although it was difficult to determine the most effective approach due to the heterogeneity of studies [9]. Despite growing global attention to stigma toward people with mental illness, important research gaps remain in low- and middle-income countries (LMICs) [9]. Most evidence on stigma-reduction interventions among healthcare professionals and medical students has been generated in high-income countries, where mental health services, educational resources, and cultural perceptions of mental illness differ substantially from those in LMICs. Consequently, the transferability of these interventions to LMIC settings remains uncertain. In many LMICs, including countries in North Africa and the Middle East, mental illness continues to be strongly influenced by cultural beliefs, social norms, and structural barriers within health systems, which may shape both the manifestation of stigma and the way future healthcare providers interact with people with mental illness [14]. Furthermore, mental health training within medical curricula in LMICs often remains limited, with fewer opportunities for structured contact with people with mental illness and for training that explicitly addresses stigma and discrimination in clinical practice. In Tunisia specifically, research on mental health stigma has largely focused on public attitudes or patient experiences, while little attention has been given to stigma among medical students or to the evaluation of structured anti-stigma interventions within medical education [15]. A study by Saguem (2023) [16] aimed to evaluate the effectiveness of a four-session educational intervention for reducing stigma of mental illness targeting family medicine trainees in Tunisia. While this was a controlled study with a 2 month follow up measure, no behavioral outcomes have been assessed. As a result, there is little empirical evidence on how future physicians in Tunisia perceive mental illness, how prepared they are to respond to discrimination experienced by patients, and whether targeted educational interventions can effectively reduce stigma in this context. Addressing this gap is essential to inform context-appropriate strategies for integrating stigma-reduction approaches into medical training and ultimately improving the quality of mental health care in Tunisia.

In the light of the gaps in the literature and the field implementation challenges, the READ (Responding to Experienced and Anticipated Discrimination) anti-stigma training was developed to teach medical students how to respond constructively to experienced and anticipated discrimination among patients, as well as to reduce stigma. It was implemented at thirteen sites across ten countries including Tunisia, and aimed to assess both knowledge, attitudes, and behaviour changes among medical students [17].

This paper aims to evaluate the effectiveness of the READ training in Tunisia on mental health stigma related knowledge, attitudes, and skills in responding to patients anticipated and experienced discrimination, in a large sample of Tunisian medical students.

## Materials and methods

### Design

The Tunisian READ study was designed as an extension at one site of a non-randomized controlled study and was part of the international multisite READ study that included 13 sites in 10 low-, middle- and high- income countries. It took place at the only psychiatric specialist hospital in Tunisia. The control group was necessary to control for the possible effects of completing the baseline measures and the psychiatry rotation. Baseline assessments were conducted after written informed consent was obtained and prior to the implementation of the READ training. Follow-up assessments were repeated at the end of the psychiatric clinical rotation, after completion of the second READ training session. The interval between baseline and follow-up measurements ranged from two to three weeks, depending on the duration of the psychiatric rotation, which lasted between three and four weeks. Students in the control group followed the same assessment procedures at baseline and follow-up but did not receive the READ training. For a more detailed description of the READ study please see protocol ([17]).

### Study and sample characteristics

Included participants were final year medical students from the Faculty of Medicine of Tunis undertaking their psychiatric rotation at the department “Psychiatry A”. The recruitment period extended from March 13^th^, 2017 to December 20^th^, 2019. Students that did not sign the consent form were excluded from the study.

Intervention and control groups entered the study consecutively as part of their psychiatry rotation. The control group completed all the baseline and follow-up measures but received only feedback on the OSCE.

### Outcome measures

Measures for the study were used in French language, as medical education and communication between health professionals in clinical settings in Tunisia are conducted in French. However, role plays were performed in the Tunisian dialect, which is the language commonly used with patients in daily practice.

#### Knowledge.

Knowledge was measured by the Mental Health Knowledge Schedule (MAKS) [18]. This 12 items scale includes six items inquiring about stigma-related mental health knowledge areas: help-seeking, recognition, support, employment, treatment, and recovery, and six items that question students’ classification of various conditions as mental illnesses. The total score is calculated based on item 1–6. Items 7–12 were not used in our study to reduce the burden of the questionnaires and because these items are more suitable for use in general population samples, for which the measure was designed. Higher MAKS scores indicate greater knowledge. The initial validation of the MAKS was carried out in an English general population sample with the MAKS test-retest reliability being moderate to substantial (Lin’s concordance statistic = 0.71) and the overall internal consistency among items 1–6 being moderate (Cronbach’s alpha = 0.65) [19].

For our study, the MAKS was translated by a bilingual translator; and the adaptation was done together with a group of experts (psychiatrists; psychiatry residents, psychologists, psychiatry nurses). Subsequently (in 2023), a validation study of the Arabic version of the MAKS was carried out in a sample of Tunisian university students showing satisfactory psychometric properties (ref Ben Amor M et al).

#### Attitudes.

Attitudes to mental illness and psychiatry were measured with the Mental Illness Clinicians’ Attitudes version 2 for medical students (MICA2) [20]. This 16-item scale evaluates discriminating attitudes to both PWMI and to psychiatry as a medical specialty. It was developed through focus groups with medical students and psychiatry residents, service users, and carers, and tested with medical students. Lower MICA2 scores indicate less stigmatizing negative attitudes towards PWMI. The original MICA2 shows good psychometric properties.

Both MICA2 test-retest reliability and internal consistency were good (Lin’s concordance statistic = 0.80 and Cronbach’s alpha = 0.79 respectively) [20].

The Tunisian study used a French translated version of MICA2 which was already available on the International Study of Discrimination and Stigma Outcomes (INDIGO) website (http://www.indigo-group.org/). Also, both questionnaires (MAKS and MICA2) were pilot-tested in the target group.

#### Skills.

Skills were assessed via an OSCE (Observed Structured Clinical Examination), which is a one-to-one based examination, that usually relies on five to ten minutes-long clinical stations, using real or simulated patients, and rated by impartial examiners [21]. The READ initial scenario (https://indigo.group.org/research/indigo-network/indigo-read-responding-to-experienced-and-anticipated-discrimination/) was adapted to the Tunisian context: a Tunisian medical student, doing his rotation at a primary healthcare center, was approached for advice by a service user recently diagnosed with mental illness. The service user, a bank agent, consulted the general practitioner of the center which made the diagnosis of psychosis and referred him for specialised treatment at “Razi” Hospital. The simulated patient reported experienced and anticipated discrimination, and the student had to acknowledge it, show an empathic attitude, explore the service user’s concerns, and express support. The OSCE has been constructed specifically to assess behaviour and communication skills. The psychiatry resident co-organizing the study performed the patient role using the adapted Tunisian case scenario. The OSCE role play was done in Tunisian Arabic to be closer to “real world experience” and the psychiatry resident was given a briefing sheet to standardize his/her presentation and responses to students. The student was assessed by both the simulated patient and an observer, the senior psychiatrist, on (i) their response to the reports of anticipated and discrimination; and (ii) the extent to which they acknowledged the stereotypes of people with psychosis and distinguished it from the diagnosis and proposed treatment. The OSCE score for each student was entered by the observing senior psychiatrist at the end of the role plays as “clear pass”, “borderline pass”, “borderline fail”, or “clear fail”.

### READ training development and delivery

READ training was developed at King’s College London (KCL) with medical students as its main target group [17]. It aimed to i) enhance their ability to improve patients’ quality of life such as access to employment opportunities and medical care, ii) combat discrimination and apply evidence-based anti-stigma interventions, and iii) reduce behaviours that might be perceived as discriminatory by patients. The training focused on the two following key elements that proved their effectiveness in other programmes [22]: a) Co-delivery of the training by a psychiatrist and a person with lived experience of mental illness and brief filmed testimonies by the latter to ensure multiple forms of contact, and b) testimonies by the person with lived experience focused on recovery. The adoption of contact as the main anti-stigma approach in the READ study was based on some of Knaak et al’s conclusions on the main key ingredients for effective changes in attitudes, whether for face-to-face or video-based interventions [22]: i) an enthusiastic facilitator, ii) social contact with an expert with lived experience, iii) focus on behavioural changes and guidance on what to say and what to do, and tackling myths and misconceptions.

The training was provided over two sessions to students in groups of 10–12 students to allow for questions and to feedback after role plays: 1) a first session of 1.5 hours near the start of the psychiatry rotation based on face to face or filmed testimony by the expert by experience about his mental illness, slide presentations about stigma and its impact, and two role plays, one of experienced discrimination in personal life and one of anticipated discrimination in the professional field, and 2) a second session of one hour one week later and before the end of the rotation that served as a catalyst to the training’s outcomes by allowing time for medical students to observe and identify incidents of patients’ experienced or anticipated discrimination within the department that they discussed in the second session [23]. This session sensitized students to internalized stigma of patients with mental illness through role plays where students acted as a general practitioner that tries to convince a patient with a first-episode psychosis to have a psychiatric follow-up. It also tackled stigma of mental illness by health professionals to reflect upon its impact on professional abilities and patient care. The manual and all intervention materials are available at the INDIGO website (see https://www.indigo-group.org/indigo-projects/project-4-improving-knowledge-attitudes-and-behaviour-of-medical-students-towards-people-with-mental-illness-lead-dr-claire-henderson/)

### Procedures

An information session by the senior psychiatrist and the psychiatry resident, at which students were given the information sheets, was conducted in the first week of the rotation and at least 24 hours before the first session.

Once written informed consent was obtained, participating students in the intervention group were given baseline MAKS and MICA2 to complete and then undertook baseline OSCE. The OSCE was performed by the psychiatry residents in the presence of the senior psychiatrist.

Data collection of the follow-up measures (MAKS, MICA2, and OSCE) was repeated at the end of the clinical rotation and after the completion of the second session. The time between the baseline and follow-up data collection for the intervention and the control groups ranged from two to three weeks depending on the length of the psychiatric rotation (three to four weeks).

The control groups followed the same procedure and completed the same measures at baseline and follow-up but didn’t receive the READ training.

Participants that missed one of the sessions for a non-voluntary reason were debriefed about its content afterwards and still included in the study.

### Statistical analyses

Statistical analyses were performed using Stata /IC.16 for data management and analysis. Percentages were calculated for the categorical variables student gender and OSCE, and means (+/- SD) were calculated for the continuous variables student (age, MAKS, and MICA2) for both intervention and control groups. Parametric t-test for paired groups were performed to compare the mean scores of the MAKS and MICA2, and chi-square tests were performed to compare the percentages of the OSCE categories between the intervention and control group before and after the intervention.

All participants had their MAKS and MICA2 scores rated and entered in the database except for some participants lacking both their baseline and follow-up OSCE scores. We used a parametric t-test and a chi-square test to look for differences in age and gender respectively between the group of participants missing their OSCE and the rest of the sample with completed OSCE scores.

Linear regression models were used to test for the association between the outcome measures (MAKS, MICA2) with the intervention/control variable unadjusted and after adjusting for demographic variables (student age and gender) and MAKS and MICA2 baseline outcome scores. Due to low numbers of participants in some categories of the OSCE, participants who scored “clear fail” and “borderline fail” were grouped under the category “fail”, and participants who scored “clear pass” and “borderline pass” were grouped under the category “pass”.We used a parametric t-test and a chi-square test to look for differences in age and gender respectively between the group of participants missing their OSCE and the rest of the sample with completed OSCE scores. A logistic regression model was used to explore the effect of READ on the follow-up OSCE scores. An adjusted Odd Ratio (OR) and corresponding 95% confidence intervals (CI) was calculated after adjusting for gender, age, and OSCE baseline.

### Ethical considerations

The INDIGO- READ multisite original project had approval from King’s College London Psychiatry, Nursing and Midwifery Research Ethics Subcommittee (reference LRS-15/16–2894). The INDIGO- READ Tunisian site approval was obtained from Razi Hospital Ethics Committee on January 20, 2017, with an extension granted on March 13, 2018 (reference RPA1/2018). All participants gave oral and written consent. Data were collected and treated anonymously.

## Results and discussion

**Fig 1:**
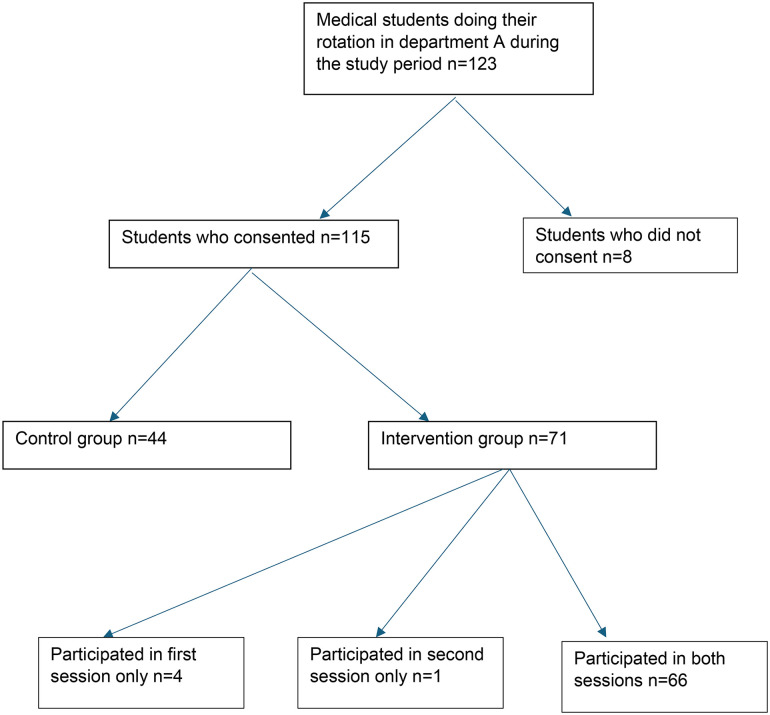
Study flow chart.

From April 2017 to December 2019, 123 final-year medical students undertook their psychiatric rotation at the department “Psychiatry A” of Razi Hospital. Among these students, eight did not consent to participate in the study (Fig 1). Our sample was, therefore, composed of 115 students (44 controls and 71 intervention). Most students were female (74.78% vs 25.22%) which corresponds to the current gender distribution at the Faculty of Medicine of Tunis. They were aged between 21 and 24 years old (mean age: 23 years old + /- 0.72) (Table 1). Five students did not attend either the first or the second presentation, however, they were then debriefed about its contents and still included in the READ study (Fig 1).

**Table 1:** Demographic characteristics.

Gender	Control (n = 44)	Intervention (n = 71)	Total (n = 115)
Male	12 (27.27%)	17 (23.94%)	29 (25.22%)
Female	32 (72.73%)	54 (76.06%)	86 (74.78%)
Age	Control (mean + /- sd)	Intervention (mean + /- sd)	Total (mean + /- sd)
	23.11 + /- 0.93	22.95 + /- 0.57	NA

There was no evidence of an association between the explanatory variable (intervention or control) and gender (chi-square test, p = 0.69) or age (t-student test, p = 0.26) (Table 1).

Table 2 shows the MAKS and MICA2 mean total scores and Table 3 the OSCE categories percentages at baseline for the control and intervention arms.

**Table 2:** Mean scores (MICA2 and MAKS), and percentages (OSCE) for each study arm at baseline and follow-up.

Control (n = 44)			Intervention (n = 71)		
Pre	Post	p-value	Pre	Post	p-value
n = 44 20.93 (3.03)	n = 44 22.70 (2.21)	<0.001	n = 71 21.63 (2.20)	n = 71 23.84 (2.62)	<0.001
n = 44 44.90 (8.97)	n = 44 41.40 (8.50)	0.008	n = 71 43.73 (7.76)	n = 71 33.52 (9.56)	<0.001

**Table 3:** Percentages (OSCE) for each study arm at baseline and follow-up.

	Control (n = 44)		Intervention (n = 71)		Total (n = 115)	
	Pre	Post	Pre	Post	Pre	Post
Fail	16 (36.36%)	7 (15.91%)	29 (40.85%)	5 (7.04%)	44 (38.26%)	12 (10.43%)
Pass	12 (27.27%)	21 (47.73%)	16 (22.54%)	40 (56.34%)	28 (24.35%)	61 (53.04%)
Missing	16 (36.36%)	16 (36.36%)	26 (36.62%)	26 (36.62%)	43 (37.40%)	42 (35.52%)

At follow up and compared to the baseline, both intervention and control groups had higher MAKS mean total scores (from 20.93; SD = 3.03 to 22.70; SD = −2.21 for the control group, and from 21.63; SD = 2.19 to 23.84; SD = −2.62 for the intervention group) and lower MICA2 mean total scores (from 44.90; SD = −8.97 to 41.40; SD = −8.50 for the control group; and from 43.73; SD = −7.76 to 33.52; SD = −9.56 for the intervention group) (Table 2).

The percentages of participants that scored” pass” at OSCE at the follow-up increased for both groups (from 27.27% to 47.73% for the control group; and from 22.54% to 56.34% for the intervention group) (Table 3).

In our sample, OSCE data were missing for 73 participants (63.5% of the total sample, N = 115). Analyses were conducted on complete cases (n = 42). No imputation was performed. Given the extent of missing data, findings should be interpreted with caution due to potential bias. We didn’t find differences in age (p = 0.73) and gender (p = 0.53) between the groups with and without OSCE scores. There were no missing data from MAKS and MICA2 scores.

Male students were more likely to be scored “pass” at OSCE than female students (p = 0.003).

There was no evidence of a difference between MAKS mean total scores of the intervention and control group after adjusting for age, gender, and baseline MAKS mean total scores (Adjusted coefficient:0.70; p = 0.11; 95% CI: −0.16 to 1.6) (Table 2).

On the other hand, after the READ training, the adjusted coefficient of MICA2 mean total scores in the intervention group was lower than the adjusted coefficient of the control group after adjusting for age, gender, and MICA2 baseline scores (Mean difference (MD): −7.88 p < 0.001; 95% CI: −10.23 to −3.96) (Table 2).

After the READ training, the intervention group was 4.45 times more likely to be scored “pass” at OSCE compared to the control group (p = 0.046; 95% CI: 1.03 to 19.26) after adjusting for age, gender, and OSCE baseline scores (Table 4).

**Table 4:** Associations between the READ intervention and MAKS MICA2 and OSCE.

	Unadjusted MD Cohen’s D	95% CI	p-value	Adjusted MD**	95% CI	p-value
MAKS	1.14	0.2 to 2.08	0.02*	0.70	−0.16 to 1.6	0.11
	0.45	0.08 to 0.81		0.27	−0.06 to 0.63	
MICA2	−7.88 −0.95	−11.37 to −4.40 −1.37 to −0.53	<0.001*	−7.10 0.86	−10.23 to −3.96 −1.24 to −0.48	<0.001*
	Unadjusted OR	95% CI	p-value	Adjusted OR***	95% CI	p-value
OSCE	2.66	0.75 to 9.43	0.12	4.45	1.03 to19.26	0.046*

Mean Differences (MD), Cohen’s D and Odds Ratio (OR) and corresponding 95% CIs are presented in the table.

* Significance level: p < 0.05

**after adjusting for age, gender, and corresponding baseline scores.

***after adjusting for age, gender, and corresponding OSCE scores.

CI: Confidence Interval, MAKS: Mental Health Knowledge Schedule, MICA2: Mental Illness Clinicians’ Attitudes version 2, OR: Odd Ratio, OSCE: Observed Structured Clinical Examination, RC: Regression Coefficient.

### Regression analysis

Using regression models to examine the association between the outcome measures and demographic variables, student gender at baseline was the only significant predictor for OSCE, male students being more likely to be scored “pass” at OSCE than female students (p = 0.003).

There was no significant difference between MAKS mean total scores of the intervention and control group after adjusting for age, gender, and baseline MAKS mean total scores (Adjusted coefficient:0.70; p = 0.11; 95% CI: −0.16 to 1.6) (Table 2).

On the other hand, after the READ training, the adjusted coefficient of MICA2 mean total scores in the intervention group was 7.88 lower than the adjusted coefficient of the control group after adjusting for age, gender, and MICA2 baseline scores (p < 0.001; 95% CI: −10.23 to −3.96) (Table 2).

After the READ training, the intervention group was 4.45 times more likely to be scored “pass” at OSCE compared to the control group (p = 0.046; 95% CI: 1.03 to 19.26) after adjusting for age, gender, and OSCE baseline scores (Table 4).

## Discussion

READ-Tunisia is the first study investigating the effectiveness of anti-stigma training in reducing the stigma of medical students towards PWMI and including both knowledge, attitudes, and behaviours as its measured outcomes in the Eastern Mediterranean region. Complying to the standards of the international multisite READ study, it used a rigorous methodology through the implementation of a manualized anti-stigma training based on theoretical foundations and medical education groundwork [17]. The READ training proved its effectiveness in decreasing students’ stigmatizing attitudes and improving their skills while it had no effect on their stigma mental health-related knowledge beyond the impact of the rotation.

This is in accordance with findings from the review done by Thornicroft et al in 2016 that revealed a consistent pattern across the literature of attitudinal and behavioural changes and less evidence for knowledge improvement in the immediate aftermath of anti-stigma trainings [7]. A 2022 systematic review carried by Gervas et al [24] found few cases of knowledge gain in interventions based on methods such as teaching practices, social contact, direct contact, contact with recovered patients, educational emails, filmed contact or in vivo exposure. The absence of significant changes in knowledge gain in our study might be due to the small sample size. Indeed, in the large international multisite READ study, anti-stigma training was associated with positive changes in knowledge, attitudes, skills, and patient perceived empathy among medical students [25]. However, another INDIGO site study done in the Czech Republic with smaller sample size than our study demonstrated reductions in stigma-related attitudes and improvements in mental illness knowledge [26]. Another explanation might be the good pre-acquired knowledge about mental illness during medical education among our sample. Indeed, medical students undertaking their studies in the Faculty of Medicine of Tunis are introduced to mental illness during their second year of medical studies where they undertake a two-week psychiatric rotation at the hospital, along with teaching about the psychopathology of mental illness at the faculty. In their fifth year, they are taught a whole module about the different psychiatric disorders, usually before or during the psychiatric rotation. However, these teachings focus on psychopathological and clinical aspects of psychiatric disorders and do not aim to increase awareness about the stigma of mental illness and its consequences on patients and their families. More than that, didactic methods are predominantly based on traditional lecturing where students passively acquire knowledge about mental illness without learning how to behave with patients.

Instead, READ training allocates most of its time to interactive and self-reflective discussions and role plays where students are either playing the role of a patient with anticipated and experienced discrimination or the role of a health professional/friend with the task of responding to discrimination. In both cases, students are confronted with their potential stigmatizing attitudes and work towards acquiring the necessary anti-stigma skills.

Our findings contribute to the growing body of evidence supporting the effectiveness of structured anti-stigma interventions implemented during psychiatric training in LMIC contexts. However, it is also important to consider whether such interventions lead to sustained long-term improvements. In a systematic review by Gervás et al [24], the authors reported that many direct-contact interventions did not demonstrate long-term benefits, highlighting the need to better understand factors influencing the durability of anti-stigma effects. In particular, the duration and intensity of the intervention may play an important role in determining its impact. Longer or repeated anti-stigma training sessions may allow for deeper reflection and consolidation of learning, potentially leading to greater and more sustained improvements in knowledge, attitudes, and behavioural outcomes. Future studies should therefore explore whether integrating repeated sessions or longitudinal training components within medical curricula could enhance the sustainability and magnitude of anti-stigma intervention effects.

Previous studies investigating the impact of psychiatric rotations on students and undergraduates in the health field found mixed results, with some suggesting a worsening of misconceptions and stereotypes about PWMI [7, 9]. A Greek study found that final-year medical students held more stigmatizing attitudes towards patients with schizophrenia at the end of their psychiatric rotation [27]. An Egyptian interventional study including 300 fifth-year medical students from Ain Shams University found no change in their MICA scores after three weeks of training [28]. In both studies, students were given training on common psychiatric disorders, interviewing skills, psychotherapy, and pharmacotherapy. The outcomes of the studies were attributed to insufficient duration of the psychiatric rotation. However, evidence regarding the relationship between the duration of psychiatric training and stigma reduction remains inconclusive. While some studies suggest that three-week rotations may be insufficient to meaningfully influence students’ perceptions [29], others have reported improvements in knowledge, beliefs, and attitudes following rotations lasting more than four weeks [30–32], and even stronger effects with longer educational programs, such as 13-week interventions that significantly reduced social distance toward PWMI [30]. Variations in findings could be explained by other factors such as differences in the studies’ designs, the implementation strategies, the used outcome measures, but also the conditions of the clinical rotations [9]. In LMICs like Tunisia and Egypt where access to mental health services is difficult and health coverage for psychiatric disorders is insufficient, medical students undertaking their psychiatric rotations in centralized psychiatric hospitals tend to witness only patients with severe symptoms. Consecutively, students may acquire biased negative perceptions about mental illness [33].

### Strengths and limitations

The Tunisian READ study is the first interventional study investigating the effectiveness of anti-stigma training in improving future doctors’ knowledge, attitudes, and behaviours towards PWMI in Tunisia. Given the dearth of findings from LMICs in this topic, our results may lead the pathway for further primary and tailored research to low and middle-income settings.

The READ study is among the few studies that measured the impact of anti-stigma training on participants’ behaviours by including skills acquisition as one of its main outcome measures. Medical education research has shown that improving interviewing and communication skills of medical students has the benefit of decreasing their discomfort and distress while dealing with psychiatric symptoms [34]. Consequently, they will have more positive responses and PWMI will feel more comfortable talking about experienced and anticipated discrimination [35].

The use of internationally used scales will allow for future comparisons of like-with-like outcomes with the results of the other READ study sites.

The rate of participation in the study was high (95% of the students undertaking their psychiatric rotation in the department during the study agreed to participate) with no dropouts.

However, like most of the previous studies, the Tunisian READ study presents weaknesses in its methodological approach: first, the non-randomization of participants might have introduced a self-selection bias in our study. Although groups were pre-designated by the Medical Faculty of Tunis, students with more stigmatizing attitudes and behaviours may have been more likely to not consent to the study if the group was chosen as an intervention group while students with less prior stigmatizing attitudes and behaviours may have been more likely to participate.

Although our study included more participants compared to the other sites, our sample size might have not been large enough to generate enough power and detect a significant improvement in knowledge gain between the intervention and the control group. We were also unable to perform the mediation analysis to explore the role of anxiety reduction and increased empathy in this sample, both of which were found to be important in the multisite study [25].

The versions of the MICA and MAKS administered during the study were translated to French but not validated for use in the Tunisian context, which might have decreased their ability to correctly assess participants’ knowledge and attitudes.

Corrigan et al largely demonstrated that live contact was more effective than filmed contact [5].However, because it was difficult to find a patient with lived experience who was available, well, and eager to share their personal experience with all intervention groups, some groups benefited from a live contact while others watched a recorded testimony. Hence, using live contact for all groups might have resulted in greater effect of the intervention on the different outcomes since medical students are able to interact with persons with lived experience.

It is also important to consider that some participants didn’t attend the full training and were still included in the study. Although they were debriefed about the content of the session, they were not able to participate in some of the role plays and benefit from all the READ training components. This might have underestimated the effect of the intervention on the overall intervention’s group attitudinal and behavioural improvement.

The OSCE role plays were played and rated by the same investigators that led the presentations and conducted the whole study which might have introduced some rater’s bias in the OSCE results.

The OSCE itself lasted between five to seven minutes per student which, according to the psychiatry resident who acted the role play, didn’t provide the participants with enough time to explore the different areas that were required to achieve a good score.

### Implications

The Tunisian READ training has proven to be feasible and effective in changing medical students’ attitudes and behaviors on the short-term. The results of our study could serve to advocate among stakeholders to integrate it or comparable interventions in the Tunisian medical curriculum and widely implement it in the regular training of medical students in Tunisia. However, longer follow-up is needed to confirm the sustainability of our results and their implementation in practice on the long-term. Further research will help to understand better the specificities of our context and the background of our target population to increase the impact of our intervention. It is also necessary to determine the active ingredients and the causal pathways of this positive relationship so we can fully understand the influence of the READ training on knowledge, attitudes, and behaviours towards PWMI.
