# Supplementary material for: Effectiveness of the Responding to Experienced and Anticipated Discrimination (READ) training on reducing stigma for medical students in Tunisia
Source: PLoS One. 2026 Jun 29;21(6):e0352158. doi: 10.1371/journal.pone.0352158 (PMC13313333; doi:10.1371/journal.pone.0352158)
Supplement: Attachment — Submitted filename: Response to reviewers.docx [file pone.0352158.s002.docx]

Effectiveness of the Responding to Experienced and Anticipated Discrimination (READ) training on reducing stigma for medical students in Tunisia

Thank you for this detailed and constructive review. Please find our answers point by point.

Areas for Improvement / Major Revisions

1. Background and justification:

o The research problem could benefit from more support from the literature. Some key studies mentioned in the introduction are cited without details or examples. Expanding the literature review would better justify the need for the study.

We have extended the literature review by adding more recent studies, in particular the scoping review by Chen et al (2024) about Interventions to decrease health students' stigma toward schizophrenia.

We also point to the different types and durations of anti-stigma interventions. Finally, stigma-reduction interventions specifically among medical students were briefly described.

o The research gap is not clearly defined. Authors should explicitly state what is missing in prior research, especially in the context of LMICs and Tunisia.

Thank your suggestion. We have now clearly defined the research gap in LMICs and specifically Tunisia by adding one paragraph (lines 70 -94)

Despite growing global attention to stigma toward people with mental illness, important research gaps remain in low- and middle-income countries (LMICs) [[7]](https://paperpile.com/c/IMtldE/97CYo). Most evidence on stigma-reduction interventions among healthcare professionals and medical students has been generated in high-income countries, where mental health services, educational resources, and cultural perceptions of mental illness differ substantially from those in LMICs. Consequently, the transferability of these interventions to LMIC settings remains uncertain. In many LMICs, including countries in North Africa and the Middle East, mental illness continues to be strongly influenced by cultural beliefs, social norms, and structural barriers within health systems, which may shape both the manifestation of stigma and the way future healthcare providers interact with people with mental illness [8]. Furthermore, mental health training within medical curricula in LMICs often remains limited, with fewer opportunities for structured contact with people with mental illness and for training that explicitly addresses stigma and discrimination in clinical practice. In Tunisia specifically, research on mental health stigma has largely focused on public attitudes or patient experiences, while little attention has been given to stigma among medical students or to the evaluation of structured anti-stigma interventions within medical education [9]. As a result, there is a lack of empirical evidence on how future physicians in Tunisia perceive mental illness, how prepared they are to respond to discrimination experienced by patients, and whether targeted educational interventions can effectively reduce stigma in this context. Addressing this gap is essential to inform context-appropriate strategies for integrating stigma-reduction approaches into medical training and ultimately improving the quality of mental health care in Tunisia.

2. Methodology:

o The psychometric properties of the instruments (MAKS and MICA2) are not described. Including validity, reliability, and prior use in similar populations would strengthen the methodology section.

The psychometric properties of the MAKS and MICA as well as their use in specific populations were added.

o The intervention was adapted to the Tunisian context, and the role-plays were conducted in Tunisian Arabic, while instruments were translated into French. This could be clarified further to ensure readers understand how linguistic and cultural adaptations were handled.

Thank you for your remark. Indeed, we have now further clarified why the role-plays were conducted in Tunisian Arabic while instruments were translated to French We have explained that medical education and communication between health professionals in clinical settings in Tunisia are conducted in French whereas the language commonly used with patients in daily practice is Tunisian Arabic, hence the use of Tunisian Arabic for the role plays.

o The study design mentions baseline and follow-up measurements, but the timing and potential longitudinal follow-up are not fully detailed. It is recommended to clarify whether a true follow-up design was implemented.

We have clarified the timing of baseline and follow-up measurements in the Methods section. Baseline assessments were conducted prior to the intervention, and follow-up assessments were repeated at the end of the psychiatric rotation after completion of the second READ session. The interval between measurements ranged from two to three weeks depending on the duration of the rotation. We have also clarified that the study employed a short-term pre-post follow-up design rather than a long-term longitudinal follow-up.

o Effect size calculations are not reported. Providing effect sizes (Cohen’s d or odds ratios) for primary outcomes would help interpret the magnitude of the intervention’s impact.

To improve clarity, we have now revised the manuscript to make the reporting of these effect size measures more explicit in the corresponding Table 4.

3. Discussion:

o Some points are well argued, but additional references, particularly from other sites within the READ project or similar multisite studies, would enhance credibility and allow readers to explore related research.

We have mentioned in the discussion another INDIGO READ site study done in the Czech Republic mentioning the outcomes which partly align with our study (Janoušková et al, 2019) (lines 343-345).

We have also enriched the discussion using the literature review 2022 by Gervas et al about the Effectiveness and types of interventions to reduce mental illness-related stigma among Medical university students (lines 337-339).

o The discussion does not mention the possibility that the sample might benefit from longer or repeated sessions to achieve greater impact. Including this consideration would provide a more balanced

Interpretation.

We fully agree with this comment and therefore added a paragraph in the discussion (lines 363-372) suggesting that longer or repeated anti-stigma training sessions may allow for deeper reflection and consolidation of learning, potentially leading to greater and more sustained improvements in knowledge, attitudes, and behavioural outcomes. We also added that future studies should therefore explore whether integrating repeated sessions or longitudinal training components could enhance the sustainability of the intervention.

Minor Comments

• Consider clarifying missing OSCE data and how it was handled in the analysis.

We have added the following at lines 285-287:

In our sample, OSCE data were missing for 73 participants (63.5% of the total sample, N = 115). Analyses were conducted on complete cases (n = 42). No imputation was performed. Given the extent of missing data, findings should be interpreted with caution due to potential bias.

• Ensure consistent formatting of tables and figures for better readability.

The tables have been formatted according to the journal guidelines.

• Provide more detail on the intervention’s content (e.g., role-play scenarios, live vs. video testimonials) for reproducibility.

Thank you for this suggestion. As the article is already quite long, we have indicated where to find all the documents for the intervention online. We have provided the link to the materials adding this sentence at line 209: The manual and all intervention materials are intervention materials are available at the INDIGO website (see https://www.indigo-group.org/indigo-projects/project-4-improving-knowledge-attitudes-and-behaviour-of-medical-students-towards-people-with-mental-illness-lead-dr-claire-henderson/)
