# Supplementary material for: Effectiveness of the Responding to Experienced and Anticipated Discrimination (READ) training on reducing stigma for medical students in Tunisia
Source: PLoS One. 2026 Jun 29;21(6):e0352158. doi: 10.1371/journal.pone.0352158 (PMC13313333; doi:10.1371/journal.pone.0352158)
Supplement: Peer review history

# Peer review: Decision Letter 0

**DOI:** 10.1371/journal.pone.0352158.r001

**Authors:** Lorenzo Faggioni

16 Feb 2026

PONE-D-25-33052 Effectiveness of the Responding to Experienced and Anticipated Discrimination (READ) training on reducing stigma for medical students in Tunisia PLOS One

Dear Dr. Henderson,

Thank you for submitting your manuscript to PLOS ONE. After careful consideration, we feel that it has merit but does not fully meet PLOS ONE’s publication criteria as it currently stands. Therefore, we invite you to submit a revised version of the manuscript that addresses the points raised during the review process.

Please submit your revised manuscript by Apr 02 2026 11:59PM. If you will need more time than this to complete your revisions, please reply to this message or contact the journal office at plosone@plos.org. When you're ready to submit your revision, log on to https://www.editorialmanager.com/pone/ and select the 'Submissions Needing Revision' folder to locate your manuscript file.

Please include the following items when submitting your revised manuscript:

- A letter that responds to each point raised by the academic editor and reviewer(s). You should upload this letter as a separate file labeled 'Response to Reviewers'.
- A marked-up copy of your manuscript that highlights changes made to the original version. You should upload this as a separate file labeled 'Revised Manuscript with Track Changes'.
- An unmarked version of your revised paper without tracked changes. You should upload this as a separate file labeled 'Manuscript'.

If you would like to make changes to your financial disclosure, please include your updated statement in your cover letter. Guidelines for resubmitting your figure files are available below the reviewer comments at the end of this letter.

If applicable, we recommend that you deposit your laboratory protocols in protocols.io to enhance the reproducibility of your results. Protocols.io assigns your protocol its own identifier (DOI) so that it can be cited independently in the future. For instructions see: https://journals.plos.org/plosone/s/submission-guidelines#loc-laboratory-protocols. Additionally, PLOS ONE offers an option for publishing peer-reviewed Lab Protocol articles, which describe protocols hosted on protocols.io. Read more information on sharing protocols at https://plos.org/protocols?utm_medium=editorial-email&utm_source=authorletters&utm_campaign=protocols.

We look forward to receiving your revised manuscript.

Kind regards,

Lorenzo Faggioni, M.D., Ph.D.

Academic Editor

PLOS One

Journal Requirements:

When submitting your revision, we need you to address these additional requirements.

1. Please ensure that your manuscript meets PLOS ONE's style requirements, including those for file naming. The PLOS ONE style templates can be found at

https://journals.plos.org/plosone/s/file?id=wjVg/PLOSOne_formatting_sample_main_body.pdf and

https://journals.plos.org/plosone/s/file?id=ba62/PLOSOne_formatting_sample_title_authors_affiliations.pdf

2. Please include a complete copy of PLOS’ questionnaire on inclusivity in global research in your revised manuscript. Our policy for research in this area aims to improve transparency in the reporting of research performed outside of researchers’ own country or community. The policy applies to researchers who have travelled to a different country to conduct research, research with Indigenous populations or their lands, and research on cultural artefacts. The questionnaire can also be requested at the journal’s discretion for any other submissions, even if these conditions are not met.  Please find more information on the policy and a link to download a blank copy of the questionnaire here: https://journals.plos.org/plosone/s/best-practices-in-research-reporting. Please upload a completed version of your questionnaire as Supporting Information when you resubmit your manuscript.

3. In the online submission form, you indicated that all relevant data is stored in an excel file within the corresponding author and can be shared with the journal without restriction.

All PLOS journals now require all data underlying the findings described in their manuscript to be freely available to other researchers, either a. In a public repository, b. Within the manuscript itself, or c. Uploaded as supplementary information.

This policy applies to all data except where public deposition would breach compliance with the protocol approved by your research ethics board. If your data cannot be made publicly available for ethical or legal reasons (e.g., public availability would compromise patient privacy), please explain your reasons on resubmission and your exemption request will be escalated for approval.

4. Please amend your list of authors on the manuscript to ensure that each author is linked to an affiliation. Authors’ affiliations should reflect the institution where the work was done (if authors moved subsequently, you can also list the new affiliation stating “current affiliation:….” as necessary).

5. Thank you for stating the following financial disclosure:

The author(s) received no specific funding for this work.

At this time, please address the following queries:

a) Please clarify the sources of funding (financial or material support) for your study. List the grants or organizations that supported your study, including funding received from your institution.

b) State what role the funders took in the study. If the funders had no role in your study, please state: “The funders had no role in study design, data collection and analysis, decision to publish, or preparation of the manuscript.”

c) If any authors received a salary from any of your funders, please state which authors and which funders.

d) If you did not receive any funding for this study, please state: “The authors received no specific funding for this work.”

Please include your amended statements within your cover letter; we will change the online submission form on your behalf.

6. Thank you for stating the following in the Acknowledgments Section of your manuscript:

Ouali, Bakolis and Henderson are supported by the Indigo Partnership research programme (Medical Research Council grant number MR/R023697/1). The funding body had no role in the design of the study, its data collection, analysis, and interpretation, or the writing of this manuscript. The Indigo Partnership programme is a part of the Indigo Network, a collaboration of research colleagues in over 30 countries worldwide committed to developing knowledge about mental illness-related stigma and discrimination, both in terms of their origins and their eradication. It is coordinated by the Centre for Global Mental Health, Institute of Psychiatry, Psychology and Neuroscience at King’s College London.

We note that you have provided funding information that is not currently declared in your Funding Statement. However, funding information should not appear in the Acknowledgments section or other areas of your manuscript. We will only publish funding information present in the Funding Statement section of the online submission form.

Please remove any funding-related text from the manuscript and let us know how you would like to update your Funding Statement. Currently, your Funding Statement reads as follows:

The author(s) received no specific funding for this work.

Please include your amended statements within your cover letter; we will change the online submission form on your behalf.

7. Please upload a copy of Supporting Information Figure 1 which you refer to in your text on page 23.

8. If the reviewer comments include a recommendation to cite specific previously published works, please review and evaluate these publications to determine whether they are relevant and should be cited. There is no requirement to cite these works unless the editor has indicated otherwise.

[Note: HTML markup is below. Please do not edit.]

Reviewers' comments:

Reviewer's Responses to Questions

**Comments to the Author**

1. Is the manuscript technically sound, and do the data support the conclusions?

The manuscript must describe a technically sound piece of scientific research with data that supports the conclusions. Experiments must have been conducted rigorously, with appropriate controls, replication, and sample sizes. The conclusions must be drawn appropriately based on the data presented.

Reviewer #1: Yes

Reviewer #2: Yes

**********

2. Has the statistical analysis been performed appropriately and rigorously?

Reviewer #1: Yes

Reviewer #2: Yes

**********

3. Have the authors made all data underlying the findings in their manuscript fully available?

The PLOS Data policy requires authors to make all data underlying the findings described in their manuscript fully available without restriction, with rare exception (please refer to the Data Availability Statement in the manuscript PDF file). The data should be provided as part of the manuscript or its supporting information, or deposited to a public repository. For example, in addition to summary statistics, the data points behind means, medians and variance measures should be available. If there are restrictions on publicly sharing data—e.g. participant privacy or use of data from a third party—those must be specified.

Reviewer #1: Yes

Reviewer #2: Yes

**********

4. Is the manuscript presented in an intelligible fashion and written in standard English?

PLOS ONE does not copyedit accepted manuscripts, so the language in submitted articles must be clear, correct, and unambiguous. Any typographical or grammatical errors should be corrected at revision, so please note any specific errors here.

Reviewer #1: Yes

Reviewer #2: No

**********

5. Review Comments to the Author

Please use the space provided to explain your answers to the questions above. You may also include additional comments for the author, including concerns about dual publication, research ethics, or publication ethics. (Please upload your review as an attachment if it exceeds 20,000 characters)

Reviewer #1: Thank you for the opportunity to review this work. The study was properly designed and conducted. The methodology described is reproducible and the results are reliable. The discussion was conducted correctly and the section on the limitations of the work was adequately expanded. I propose that the work be accepted without reservation.

Reviewer #2: I have carefully reviewed the manuscript entitled “Effectiveness of the Responding to Experienced and Anticipated Discrimination (READ) training on reducing stigma for medical students in Tunisia” evaluating the effectiveness of the READ anti-stigma training for medical students in Tunisia. Overall, the study addresses an important topic and provides valuable insights. Below are my detailed comments:

Strengths of the study

1. Relevance of the topic: The study addresses a crucial issue- stigma towards People With Mental Illness (PWMI) among medical students in a low- to middle-income country- which is under-researched.

2. Study design: The use of a controlled design, including intervention and control groups, with pre- and post-measures, is appropriate and robust.

3. Rigorous procedures: Ethical approvals were obtained, informed consent was secured, and participant anonymity was maintained.

4. Use of standardized instruments: The study employs internationally recognized measures (MAKS, MICA2, OSCE), allowing for comparison with other sites in the multisite READ study.

5. Integration with a multisite study: Inclusion in a larger international study strengthens the external validity and impact of the findings.

6. Clear reporting of outcomes: Statistical analyses are appropriate, and results are presented clearly, including tables showing pre- and post-intervention outcomes.

Areas for Improvement / Major Revisions

1. Background and justification:

o The research problem could benefit from more support from the literature. Some key studies mentioned in the introduction are cited without details or examples. Expanding the literature review would better justify the need for the study.

o The research gap is not clearly defined. Authors should explicitly state what is missing in prior research, especially in the context of LMICs and Tunisia.

2. Methodology:

o The psychometric properties of the instruments (MAKS and MICA2) are not described. Including validity, reliability, and prior use in similar populations would strengthen the methodology section.

o The intervention was adapted to the Tunisian context, and the role-plays were conducted in Tunisian Arabic, while instruments were translated into French. This could be clarified further to ensure readers understand how linguistic and cultural adaptations were handled.

o The study design mentions baseline and follow-up measurements, but the timing and potential longitudinal follow-up are not fully detailed. It is recommended to clarify whether a true follow-up design was implemented.

o Effect size calculations are not reported. Providing effect sizes (Cohen’s d or odds ratios) for primary outcomes would help interpret the magnitude of the intervention’s impact.

3. Discussion:

o Some points are well argued, but additional references, particularly from other sites within the READ project or similar multisite studies, would enhance credibility and allow readers to explore related research.

o The discussion does not mention the possibility that the sample might benefit from longer or repeated sessions to achieve greater impact. Including this consideration would provide a more balanced interpretation.

Minor Comments

• Consider clarifying missing OSCE data and how it was handled in the analysis.

• Ensure consistent formatting of tables and figures for better readability.

• Provide more detail on the intervention’s content (e.g., role-play scenarios, live vs. video testimonials) for reproducibility.

Overall Recommendation

This is a scientifically sound and methodologically robust study. The topic is important, the study is well-designed, and the results are compelling. However, revisions are required to:

• Strengthen the literature review and clarify the research gap

• Add psychometric details of instruments and effect sizes

**********

6. PLOS authors have the option to publish the peer review history of their article (what does this mean?). If published, this will include your full peer review and any attached files.

If you choose “no”, your identity will remain anonymous but your review may still be made public.

**Do you want your identity to be public for this peer review?** For information about this choice, including consent withdrawal, please see our Privacy Policy.

Reviewer #1: No

Reviewer #2: No

**********

[NOTE: If reviewer comments were submitted as an attachment file, they will be attached to this email and accessible via the submission site. Please log into your account, locate the manuscript record, and check for the action link "View Attachments". If this link does not appear, there are no attachment files.]

To ensure your figures meet our technical requirements, please review our figure guidelines: https://journals.plos.org/plosone/s/figures

You may also use PLOS’s free figure tool, NAAS, to help you prepare publication quality figures: https://journals.plos.org/plosone/s/figures#loc-tools-for-figure-preparation.

NAAS will assess whether your figures meet our technical requirements by comparing each figure against our figure specifications.

# Author comment: Author response to Decision Letter 1

**DOI:** 10.1371/journal.pone.0352158.r002

26 May 2026

Effectiveness of the Responding to Experienced and Anticipated Discrimination (READ) training on reducing stigma for medical students in Tunisia

Thank you for this detailed and constructive review. Please find our answers point by point.

Areas for Improvement / Major Revisions

1. Background and justification:

o The research problem could benefit from more support from the literature. Some key studies mentioned in the introduction are cited without details or examples. Expanding the literature review would better justify the need for the study.

We have extended the literature review by adding more recent studies, in particular the scoping review by Chen et al (2024) about Interventions to decrease health students' stigma toward schizophrenia.

We also point to the different types and durations of anti-stigma interventions. Finally, stigma-reduction interventions specifically among medical students were briefly described.

o The research gap is not clearly defined. Authors should explicitly state what is missing in prior research, especially in the context of LMICs and Tunisia.

Thank your suggestion. We have now clearly defined the research gap in LMICs and specifically Tunisia by adding one paragraph (lines 70 -94)

Despite growing global attention to stigma toward people with mental illness, important research gaps remain in low- and middle-income countries (LMICs) [7]. Most evidence on stigma-reduction interventions among healthcare professionals and medical students has been generated in high-income countries, where mental health services, educational resources, and cultural perceptions of mental illness differ substantially from those in LMICs. Consequently, the transferability of these interventions to LMIC settings remains uncertain. In many LMICs, including countries in North Africa and the Middle East, mental illness continues to be strongly influenced by cultural beliefs, social norms, and structural barriers within health systems, which may shape both the manifestation of stigma and the way future healthcare providers interact with people with mental illness [8]. Furthermore, mental health training within medical curricula in LMICs often remains limited, with fewer opportunities for structured contact with people with mental illness and for training that explicitly addresses stigma and discrimination in clinical practice. In Tunisia specifically, research on mental health stigma has largely focused on public attitudes or patient experiences, while little attention has been given to stigma among medical students or to the evaluation of structured anti-stigma interventions within medical education [9]. As a result, there is a lack of empirical evidence on how future physicians in Tunisia perceive mental illness, how prepared they are to respond to discrimination experienced by patients, and whether targeted educational interventions can effectively reduce stigma in this context. Addressing this gap is essential to inform context-appropriate strategies for integrating stigma-reduction approaches into medical training and ultimately improving the quality of mental health care in Tunisia.

2. Methodology:

o The psychometric properties of the instruments (MAKS and MICA2) are not described. Including validity, reliability, and prior use in similar populations would strengthen the methodology section.

The psychometric properties of the MAKS and MICA as well as their use in specific populations were added.

o The intervention was adapted to the Tunisian context, and the role-plays were conducted in Tunisian Arabic, while instruments were translated into French. This could be clarified further to ensure readers understand how linguistic and cultural adaptations were handled.

Thank you for your remark. Indeed, we have now further clarified why the role-plays were conducted in Tunisian Arabic while instruments were translated to French We have explained that medical education and communication between health professionals in clinical settings in Tunisia are conducted in French whereas the language commonly used with patients in daily practice is Tunisian Arabic, hence the use of Tunisian Arabic for the role plays.

o The study design mentions baseline and follow-up measurements, but the timing and potential longitudinal follow-up are not fully detailed. It is recommended to clarify whether a true follow-up design was implemented.

We have clarified the timing of baseline and follow-up measurements in the Methods section. Baseline assessments were conducted prior to the intervention, and follow-up assessments were repeated at the end of the psychiatric rotation after completion of the second READ session. The interval between measurements ranged from two to three weeks depending on the duration of the rotation. We have also clarified that the study employed a short-term pre-post follow-up design rather than a long-term longitudinal follow-up.

o Effect size calculations are not reported. Providing effect sizes (Cohen’s d or odds ratios) for primary outcomes would help interpret the magnitude of the intervention’s impact.

To improve clarity, we have now revised the manuscript to make the reporting of these effect size measures more explicit in the corresponding Table 4.

3. Discussion:

o Some points are well argued, but additional references, particularly from other sites within the READ project or similar multisite studies, would enhance credibility and allow readers to explore related research.

We have mentioned in the discussion another INDIGO READ site study done in the Czech Republic mentioning the outcomes which partly align with our study (Janoušková et al, 2019) (lines 343-345).

We have also enriched the discussion using the literature review 2022 by Gervas et al about the Effectiveness and types of interventions to reduce mental illness-related stigma among Medical university students (lines 337-339).

o The discussion does not mention the possibility that the sample might benefit from longer or repeated sessions to achieve greater impact. Including this consideration would provide a more balanced

Interpretation.

We fully agree with this comment and therefore added a paragraph in the discussion (lines 363-372) suggesting that longer or repeated anti-stigma training sessions may allow for deeper reflection and consolidation of learning, potentially leading to greater and more sustained improvements in knowledge, attitudes, and behavioural outcomes. We also added that future studies should therefore explore whether integrating repeated sessions or longitudinal training components could enhance the sustainability of the intervention.

Minor Comments

• Consider clarifying missing OSCE data and how it was handled in the analysis.

We have added the following at lines 285-287:

In our sample, OSCE data were missing for 73 participants (63.5% of the total sample, N = 115). Analyses were conducted on complete cases (n = 42). No imputation was performed. Given the extent of missing data, findings should be interpreted with caution due to potential bias.

• Ensure consistent formatting of tables and figures for better readability.

The tables have been formatted according to the journal guidelines.

• Provide more detail on the intervention’s content (e.g., role-play scenarios, live vs. video testimonials) for reproducibility.

Thank you for this suggestion. As the article is already quite long, we have indicated where to find all the documents for the intervention online. We have provided the link to the materials adding this sentence at line 209: The manual and all intervention materials are intervention materials are available at the INDIGO website (see https://www.indigo-group.org/indigo-projects/project-4-improving-knowledge-attitudes-and-behaviour-of-medical-students-towards-people-with-mental-illness-lead-dr-claire-henderson/)

Attachment Submitted filename: Response to reviewers.docx

# Peer review: Decision Letter 1

**DOI:** 10.1371/journal.pone.0352158.r003

**Authors:** Lorenzo Faggioni

7 Jun 2026

Effectiveness of the Responding to Experienced and Anticipated Discrimination (READ) training on reducing stigma for medical students in Tunisia

PONE-D-25-33052R1

Dear Dr. Henderson,

We’re pleased to inform you that your manuscript has been judged scientifically suitable for publication and will be formally accepted for publication once it meets all outstanding technical requirements.

Within one week, you’ll receive an e-mail detailing the required amendments. When these have been addressed, you’ll receive a formal acceptance letter and your manuscript will be scheduled for publication.

An invoice will be generated when your article is formally accepted. Please note, if your institution has a publishing partnership with PLOS and your article meets the relevant criteria, all or part of your publication costs will be covered. Please make sure your user information is up-to-date by logging into Editorial Manager at Editorial Manager® and clicking the ‘Update My Information' link at the top of the page. For questions related to billing, please contact billing support.

If your institution or institutions have a press office, please notify them about your upcoming paper to help maximize its impact. If they’ll be preparing press materials, please inform our press team as soon as possible -- no later than 48 hours after receiving the formal acceptance. Your manuscript will remain under strict press embargo until 2 pm Eastern Time on the date of publication. For more information, please contact onepress@plos.org.

Kind regards,

Lorenzo Faggioni, M.D., Ph.D.

Academic Editor

PLOS One

Reviewers' comments:

Reviewer's Responses to Questions

**Comments to the Author**

1. If the authors have adequately addressed your comments raised in a previous round of review and you feel that this manuscript is now acceptable for publication, you may indicate that here to bypass the “Comments to the Author” section, enter your conflict of interest statement in the “Confidential to Editor” section, and submit your "Accept" recommendation.

Reviewer #2: All comments have been addressed

**********

2. Is the manuscript technically sound, and do the data support the conclusions?

The manuscript must describe a technically sound piece of scientific research with data that supports the conclusions. Experiments must have been conducted rigorously, with appropriate controls, replication, and sample sizes. The conclusions must be drawn appropriately based on the data presented.

Reviewer #2: Yes

**********

3. Has the statistical analysis been performed appropriately and rigorously?

Reviewer #2: Yes

**********

4. Have the authors made all data underlying the findings in their manuscript fully available?

The PLOS Data policy requires authors to make all data underlying the findings described in their manuscript fully available without restriction, with rare exception (please refer to the Data Availability Statement in the manuscript PDF file). The data should be provided as part of the manuscript or its supporting information, or deposited to a public repository. For example, in addition to summary statistics, the data points behind means, medians and variance measures should be available. If there are restrictions on publicly sharing data—e.g. participant privacy or use of data from a third party—those must be specified.

Reviewer #2: Yes

**********

5. Is the manuscript presented in an intelligible fashion and written in standard English?

PLOS ONE does not copyedit accepted manuscripts, so the language in submitted articles must be clear, correct, and unambiguous. Any typographical or grammatical errors should be corrected at revision, so please note any specific errors here.

Reviewer #2: Yes

**********

6. Review Comments to the Author

Please use the space provided to explain your answers to the questions above. You may also include additional comments for the author, including concerns about dual publication, research ethics, or publication ethics. (Please upload your review as an attachment if it exceeds 20,000 characters)

Reviewer #2: Thank you for the careful revision of the manuscript and for providing detailed responses to the reviewers’ comments. The authors have adequately addressed the concerns raised during the review process, and the revisions have improved the clarity and overall quality of the manuscript.

I have no further major comments and consider the manuscript suitable for publication.

**********

7. PLOS authors have the option to publish the peer review history of their article (what does this mean?). If published, this will include your full peer review and any attached files.

If you choose “no”, your identity will remain anonymous but your review may still be made public.

**Do you want your identity to be public for this peer review?** For information about this choice, including consent withdrawal, please see our Privacy Policy.

Reviewer #2: No

**********

# Editor report: Acceptance letter

**DOI:** 10.1371/journal.pone.0352158.r004

**Authors:** Lorenzo Faggioni

PONE-D-25-33052R1

PLOS One

Dear Dr. Henderson,

I'm pleased to inform you that your manuscript has been deemed suitable for publication in PLOS One. Congratulations! Your manuscript is now being handed over to our production team.

At this stage, our production department will prepare your paper for publication. This includes ensuring the following:

* All references, tables, and figures are properly cited

* All relevant supporting information is included in the manuscript submission,

* There are no issues that prevent the paper from being properly typeset

You will receive further instructions from the production team, including instructions on how to review your proof when it is ready. Please keep in mind that we are working through a large volume of accepted articles, so please give us a few days to review your paper and let you know the next and final steps.

Lastly, if your institution or institutions have a press office, please let them know about your upcoming paper now to help maximize its impact. If they'll be preparing press materials, please inform our press team within the next 48 hours. Your manuscript will remain under strict press embargo until 2 pm Eastern Time on the date of publication. For more information, please contact onepress@plos.org.

You will receive an invoice from PLOS for your publication fee after your manuscript has reached the completed accept phase. If you receive an email requesting payment before acceptance or for any other service, this may be a phishing scheme. Learn how to identify phishing emails and protect your accounts at https://explore.plos.org/phishing.

If we can help with anything else, please email us at customercare@plos.org.

Thank you for submitting your work to PLOS ONE and supporting open access.

Kind regards,

PLOS ONE Editorial Office Staff

on behalf of

Dr. Lorenzo Faggioni

Academic Editor

PLOS One
